# The Predictive and Guidance Value of Signet Ring Cell Histology for Stage II/III Colon Cancer Response to Chemotherapy

**DOI:** 10.3389/fonc.2021.631995

**Published:** 2021-02-23

**Authors:** Huici Jiang, Dongxuan Shao, Peiyu Zhao, Yupeng Wu

**Affiliations:** ^1^ Department of Obstetrics and Gynecology, Shanghai Tenth People’s Hospital, Shanghai, China; ^2^ Department of Obstetrics and Gynecology, Shanghai Eighth People’s Hospital, Shanghai, China; ^3^ Department of Obstetrics and Gynecology, Suzhou Municipal Hospital, Suzhou, China

**Keywords:** signet ring cell histology, stage II/III, colon cancer, chemotherapy, survival

## Abstract

**Purpose:**

To evaluate the predictive and guidance value of signet-ring cell carcinoma for chemotherapy response in stage II/III colon cancer.

**Methods:**

Eligible patients were recruited from the Surveillance, Epidemiology and End Results (SEER) database. The differences between adenocarcinoma (AD) and SRCC groups in the incidence of patients’ demographic and clinical characteristics were analyzed by Pearson’s chi-squared (×2) test. Survival was analyzed using the Kaplan–Meier method, and the differences were determined by the log-rank test. Some Cox regression models were built to assess hazard ratios (HRs) of different variables with 95% confidence intervals (95% CIs).

**Results:**

In stage II AD, it was found that the receipt of chemotherapy had significantly 12.6% decreased risk of cancer-specific mortality (HR = 0.874, 95% CI = 0.825–0.927, P < 0.001). In stage II SRCC, however, the receipt of chemotherapy had significantly 70.00% increased risk of cancer-specific mortality (HR = 1.700, 95% CI = 1.032–2.801, P = 0.037). In stage III AD, it was found that the receipt of chemotherapy had significantly 45.3% decreased risk of cancer-specific mortality (HR = 0.547, 95% CI = 0.530–0.564, P < 0.001). In stage III SRCC, the receipt of chemotherapy had significantly 24.6% decreased risk of cancer-specific mortality (HR = 0.754, 95% CI = 0.632–0.900, P = 0.002).

**Conclusions:**

The cancer-specific survival (CSS) difference between AD and SRCC was not statistically significant in stage II colon cancer. We provided the first compelling evidence that chemotherapy should not be treated in stage II SRCC, while stage III SRCC should be treated with chemotherapy.

## Introduction

Colon cancer is one of the most common malignant tumors in clinical practice and among the leading causes of cancer-related deaths all over the world (1). The conventional adenocarcinoma (AD) characterized by glandular architecture accounts for more than 90% of cases according to the histologic analysis (2). Signet-ring cell carcinoma (SRCC), however, is a rare type of malignant dedifferentiated AD, accounts for only approximately 1% of colorectal cancer, and is defined as the presence of abundant intracellular mucin in more than 50% of its cells (3–7).

Given that SRCC is a rare disease in colon cancer and often not addressed in clinical trials, there is some debate about the prognostic value of this histologic subtype. SRCC had a distinct histologic appearance and underlying biologic behavior and some researchers reported that SRCC had higher pattern of peritoneal and ovarian metastasis and worse prognosis compared with AD (4, 5, 7–10). In addition, it is still unclear whether SRCC would influence clinical decision-making with the aggressive behavior (11–13). Stage II colon cancer with high-risk factors (including T4 status, poorly differentiated histology, vascular invasion, ileus, <12 lymph nodes examined, and neural invasion, as recommended by several treatment guidelines) and stage III cancer are usually treated with adjuvant chemotherapy after the resection of the primary tumor (14, 15).

Therefore, in this population-based study using a large cancer database, we aimed to evaluate the predictive and guidance value of SRCC for colon cancer response to chemotherapy in stage II and stage III colon cancer.

## Materials and Methods

### Study Population

The Surveillance, Epidemiology and End Results (SEER) program of the National Cancer Institute provided authoritative information on cancer statistics from 18 registries [San Francisco–Oakland, Connecticut, metropolitan Detroit, Hawaii, Iowa, New Mexico, Utah (since 1973), Seattle–Puget Sound (since 1974), metropolitan Atlanta (since 1975), Alaska, San Jose–Monterey, Los Angeles, rural Georgia (since 1992), greater California (excluding San Francisco, Los Angeles, and San Jose), Kentucky, Louisiana, New Jersey, and greater Georgia (excluding

Atlanta and rural Georgia, since 2000)], and it covered approximately 28% of the total US population (https://seer.cancer.gov/) (16).

As a retrospective population-based study, the flowchart of the patient selection was shown in **Figure S1**. Using the SEER database through SEER*Stat software V.8.3.5, we then extracted 404189 colorectal cancer patients from the SEER database. However, patients satisfied one of the following conditions were excluded from the cohort: without active follow-up; rectal primary; with unknown race; with T.N.M stages unknown or without radical surgery of the primary tumor. Given our study focused on stage II/III colon cancer, we also excluded patients with distant metastases or stage I disease. Only patients diagnosed with stage II/III colon cancer were included in the present analyses, and all the patients were divided in two groups according to the histology: AD and SRCC groups. The following clinical features were acquired: T stage (T1, T2, T3, and T4), N stage (N0, N1, and N2), age at diagnosis (years), race (white, black, and other), gender (male and female), grade (grade I/II, grade III/IV, and unknown), the receipt of chemotherapy (no/unknown or yes), and histological type (AD and SRCC).

### Statistical Analysis

The differences between AD and SRCC groups in the incidence of patients’ demographic and clinical characteristics were analyzed by Pearson’s chi-squared (×2) test. The primary outcome of the interest in the present study was the cancer-specific survival (CSS), which was calculated from the time of diagnosis to the time of death due to colon cancer. CSS was analyzed using the Kaplan–Meier method, and the differences were determined by the log-rank test.

Some Cox regression models were built to identify whether a pathological characteristic impacted the prognosis independently and assess hazard ratios (HRs) of different variables with 95% confidence intervals (95% CIs). Aimed to evaluate the predictive value of signet ring cell histology for stage II/III colon cancer response to chemotherapy, we then defined an interaction variable (combined by histology and chemotherapy). The common demographic and clinicopathological data, including T stage, N stage, age at diagnosis, race, gender, grade, the receipt of chemotherapy, and histological type were entered as covariates in univariate analyses and only those characteristics with a P value less than 0.20 in the univariable analyses would be considered as candidates for the multivariable analyses. Statistical significance was set as a two-sided P value less than 0.05. All the statistical analyses were performed using SPSS 23.0 (IBM Corporation, Armonk, NY, USA).

## Results

### Demographic and Clinical Characteristics

A total of 142,983 patients diagnosed with stage II/III colon cancer were recruited from the SEER database, including 141,281 patients (98.8%) with AC and 1,702 patients (1.2%) with SRCC, 72,796 patients (50.9%) with stage II disease, and 70,187 patients (49.1%) with stage III disease, 69,248 males (48.4%) and 73,735 females (51.6%), most of them were white (80.4%). The median age of all the patients in SRCC histology was 71 years. Among these patients, the median follow-up time was 46 months, 84,903 (59.38%) patients with stage II/III colon cancer were followed up for at least 1 year.

The differences between AS and SRCC groups in the incidence of patients’ demographic and clinical characteristics were shown in **Table 1**. It was found that SRCC histology was more likely to be related to T4 stage (P < 0.001), N2 stage (P < 0.001), and grade III/IV (P < 0.001), indicating that SRCC histology was more likely to be associated with adverse tumor pathology. We also noted that SRCC histology was more prone to white race (P < 0.001) and the receipt of chemotherapy (P < 0.001). But the difference between SRCC and AD histology in age of diagnosis (P = 0.849) and gender (P = 0.183) did not achieve statistical significance.

**Table 1:** Patient characteristics of stage II/III colon cancer.

	Patient characteristics		*P*
	AdenocarcinomaN = 141281 (%)	Signet ring cellcarcinomaN = 1702 (%)	
**T stage**			<0.001
** T1**	3131 (2.2)	24 (1.4)	
** T2**	6030 (4.3)	41 (2.4)	
** T3**	108556 (76.8)	1052 (61.8)	
** T4**	23564 (16.7)	585 (34.4)	
**N stage**			<0.001
** N0**	72305 (51.2)	491 (28.8)	
** N1**	46323 (32.8)	445 (26.1)	
** N2**	22653 (16.0)	766 (45.0)	
**Age (years)**			0.849
** ≤65**	52144 (36.9)	632 (37.1)	
** >65**	89137 (63.1)	1070 (62.9)	
**Race**			<0.001
** White**	113441 (80.3)	1460 (85.8)	
** Black**	16444 (11.6)	133 (7.8)	
** Other**	11396 (8.1)	109 (6.4)	
**Gender**			0.183
** Male**	68451 (48.5)	797 (46.8)	
** Female**	72830 (51.5)	905 (53.2)	
**Grade**			<0.001
** Grade I/II**	108073 (76.5)	112 (6.6)	
** Grade III/IV**	30560 (21.6)	1495 (87.8)	
** Unknown**	2648 (1.9)	95 (5.6)	
**Chemotherapy**			<0.001
** No/unknown**	90587 (64.1)	913 (53.6)	
** Yes**	50694 (35.9)	789 (46.4)	

### The Prognostic Value of SRCC in Stage II/III Colon Cancer

In **Table 2**, univariate and multivariate Cox analyses were conducted to evaluate the prognostic value of SRCC in stage II/III colon cancer. The univariate analysis produced seven variables that were then included in the multivariate analysis and the variable of gender was excluded. The results of multivariate analysis showed that SRCC histology was independently associated with 30.2% increased risk of colon cancer-specific mortality compared with AD histology (HR = 1.302, 95% CI = 1.196–1.417, P < 0.001, using AD histology as the reference). It was also found that higher T stage (P < 0.001), higher N stage (P < 0.001), older age (P < 0.001), black race (P < 0.001), and higher grade (P < 0.001) were more likely to be associated with worse CSS, and the receipt of chemotherapy was associated with 38.0% decreased risk of colon cancer-specific mortality (HR = 0.620, 95% CI = 0.603–0.637, P < 0.001, using no/unknown chemotherapy as the reference).

**Table 2:** Cox regression analyses of prognostic factors for CSS in stage II/III colon cancer.

Variable	Univariate analyses		Multivariate analyses	
	HR (95%CI)	*P*	HR (95%CI)	*P*
**Histology**		<0.001		<0.001
** Adenocarcinoma**			1	
** Signet ring cell** ** carcinoma**			1.302 (1.196-1.417)	
**T stage**		<0.001		<0.001
** T1**			1	
** T2**			1.449 (1.251-1.679)	<0.001
** T3**			2.929 (2.575-3.330)	<0.001
** T4**			6.716 (5.899-7.645)	<0.001
**N stage**		<0.001		<0.001
** N0**			1	
**N1**			2.201 (2.134-2.270)	<0.001
** N2**			4.079 (3.947-4.216)	<0.001
**Age (years)**		<0.001		<0.001
** ≤65**			1	
** >65**			1.482 (1.443-1.522)	
**Race**		<0.001		<0.001
**White**			1	
**Black**			1.333 (1.287-1.381)	<0.001
** Other**			0.936 (0.895-0.980)	0.005
**Gender**		0.515		
** Male**				
**Female**				
**Grade**		<0.001		<0.001
** Grade I/II**			1	
** Grade III/IV**			1.200 (1.167-1.234)	<0.001
** Unknown**			1.230 (1.134-1.334)	<0.001
**Chemotherapy**		<0.001		<0.001
** No/unknown**			1	
** Yes**			0.620 (0.603-0.637)	

### Stage II/III SRCC Response to Chemotherapy

**Figure 1:**
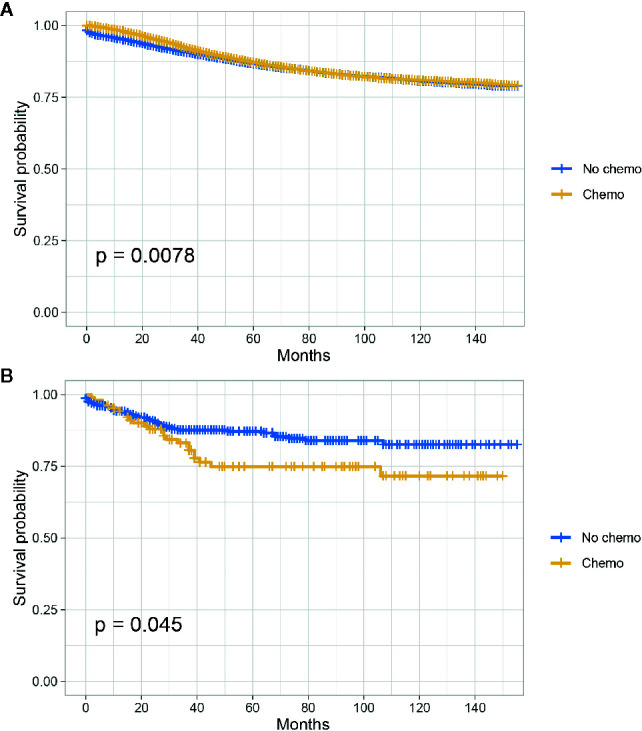
CSS among AD and SRCC patients with or without chemotherapy in stage II colon cancer. **(A)** CSS of colon cancer patients with the receipt of chemotherapy was similar to those without the receipt of chemotherapy in stage II AD; **(B)** The receipt of chemotherapy significantly decreased the CSS of colon cancer patients in stage II SRCC.

The Kaplan-Meier CSS curves comparing the survival improvement offered by chemotherapy of SRCC and AD histology in stage II colon cancer were plotted in **Figure 1**. In stage II colon cancer with the histology of AD, it was found that the CSS of colon cancer patients with the receipt of chemotherapy was similar to those without the receipt of chemotherapy though survival difference was statistically significant and the five-year CSS rates of patients with and without the receipt of chemotherapy were 86.7 and 87.2%, respectively (P = 0.0078, **Figure 1A**); in stage II colon cancer with the histology of SRCC, however, it was found that the receipt of chemotherapy significantly decreased the CSS of colon cancer patients and the 5-year CSS rates of patients with and without the receipt of chemotherapy were 74.9 and 87.2%, respectively (P = 0.045, **Figure 1B**).

In **Table 3** and **Table S1**, univariate and multivariate Cox analyses were conducted to evaluate the predictive value of histology for colon cancer response to chemotherapy. The univariate analysis produced five variables that were then included in the multivariate analysis and the variable of gender was excluded. In stage II AD, it was found that the receipt of chemotherapy had significantly 12.6% decreased risk of cancer-specific mortality (HR = 0.874, 95% CI = 0.825–0.927, P < 0.001, **Table 3**). In stage II SRCC, however, the receipt of chemotherapy had significantly 70.00% increased risk of cancer-specific mortality (HR = 1.700, 95% CI = 1.032–2.801, P = 0.037, **Table S1**). We also noted that the CSS difference between AD and SRCC was not statistically significant in stage II colon cancer (P = 0.388).

**Table 3:** Cox regression analyses of prognostic factors for CSS in stage II colon cancer.

Variable	Univariate analyses		Multivariate analyses	
	HR (95%CI)	*P*	HR (95%CI)	*P*
**T stage**		<0.001		<0.001
** T3**			1	
** T4**			2.786 (2.656-2.923)	
**Age (years)**		<0.001		<0.001
** ≤65**			1	
** >65**			1.697 (1.616-1.781)	
**Race**		<0.001		<0.001
** White**			1	
** Black**			1.414 (1.331-1.502)	<0.001
** Other**			0.910 (0.937-0.990)	0.028
**Gender**		0.262		
** Male**				
** Female**				
**Grade**		<0.001		<0.001
** Grade I/II**			1	
** Grade III/IV**			1.071 (1.015-1.129)	0.012
** Unknown**			1.291 (1.129-1.477)	<0.001
**Histology and chemotherapy**		0.003		<0.001
** Adenocarcinoma,** ** No/unknown**			1	
** Adenocarcinoma,** ** Yes**			0.874 (0.825-0.927)	<0.001
** SRCC, No/** ** unknown**			0.880 (0.659-1.175)	0.388
** SRCC, Yes**			1.497 (0.992-2.259)	0.055

**Figure 2:**
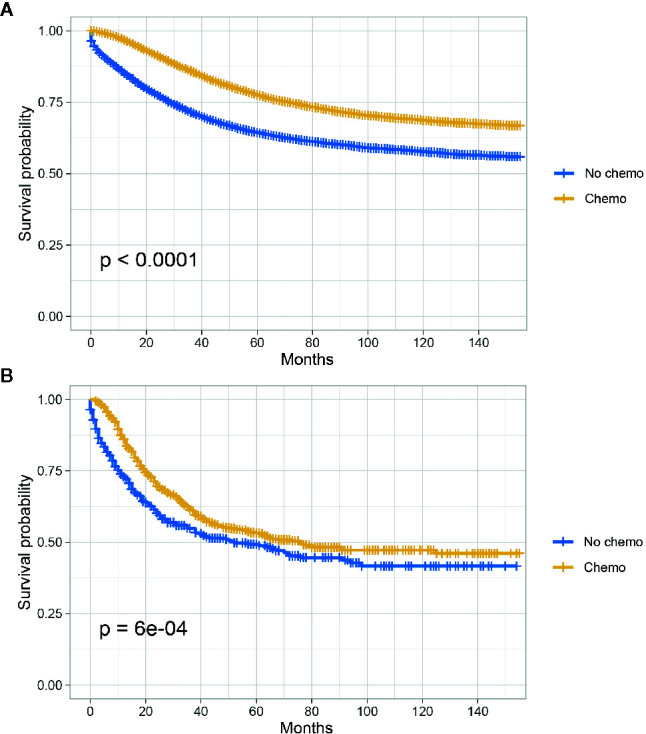
CSS among AD and SRCC patients with or without chemotherapy in stage III colon cancer. **(A)** CSS of colon cancer patients with the receipt of chemotherapy was significantly better than those without the receipt of chemotherapy in stage II AD; **(B)** The receipt of chemotherapy improved the CSS of colon cancer patients.

The Kaplan-Meier CSS curves comparing the survival improvement offered by chemotherapy of SRCC and AD histology in stage III colon cancer were plotted in **Figure 2**. In stage III colon cancer with the histology of AD, it was found that the CSS of colon cancer patients with the receipt of chemotherapy was significantly better than those without the receipt of chemotherapy and the 5-year CSS rates of patients with and without the receipt of chemotherapy were 77.5 and 64.4%, respectively (P < 0.001, **Figure 2A**); In stage III colon cancer with the histology of SRCC, it was found that the receipt of chemotherapy improved the CSS of colon cancer patients and the 5-year CSS rates of patients with and without the receipt of chemotherapy were 53.1 and 49.3%, respectively (P < 0.001, **Figure 2B**).

In **Table 4** and **Table S2**, univariate and multivariate Cox analyses were conducted to evaluate the predictive value of histology for colon cancer response to chemotherapy. The univariate analysis produced six variables that were then included in the multivariate analysis and the variable of gender was excluded. In stage III AD, it was found that the receipt of chemotherapy had significantly 45.3% decreased risk of cancer-specific mortality (HR = 0.547, 95% CI = 0.530–0.564, P < 0.001, **Table 4**). In stage III SRCC, the receipt of chemotherapy had significantly 24.6% decreased risk of cancer-specific mortality (HR = 0.754, 95% CI = 0.632–0.900, P = 0.002, **Table S2**). We also note that SRCC histology had 14.5% increased risk of cancer-specific mortality with the borderline statistical significance compared with the histology of AD (HR = 1.145, 95% CI = 0.998–1.313, P = 0.054).

**Table 4:** Cox regression analyses of prognostic factors for CSS in stage III colon cancer.

Variable	Univariate analyses		Multivariate analyses	
	HR (95%CI)	*P*	HR (95%CI)	*P*
**T stage**		<0.001		<0.001
** T1**			1	
** T2**			1.444 (1.246-1.673)	<0.001
** T3**			3.019 (2.654-3.434)	<0.001
** T4**			6.182 (5.426-7.044)	<0.001
**N stage**		<0.001		<0.001
** N1**			1	
** N2**			1.877 (1.821-1.935)	
**Age (years)**		<0.001		<0.001
** ≤65**			1	
** >65**			1.406 (1.362-1.452)	
**Race**		<0.001		<0.001
** White**			1	
** Black**			1.292 (1.237-1.350)	<0.001
** Other**			0.947 (0.897-0.999)	0.048
**Gender**		0.963		
** Male**				
** Female**				
**Grade**		<0.001		<0.001
** Grade I/II**			1	
** Grade III/IV**			1.259 (1.218-1.300)	<0.001
** Unknown**			1.189 (1.074-1.316)	0.001
**Histology and chemotherapy**		<0.001		<0.001
** Adenocarcinoma,** ** No/unknown**			1	
** Adenocarcinoma,** ** Yes**			0.547 (0.530-0.564)	<0.001
** SRCC, No/** ** unknown**			1.145 (0.998-1.313)	0.054
** SRCC, Yes**			0.863 (0.766-0.973)	0.016

## Discussion

SRCC was a very rare histological type of AD and the reported incidence ranged from 0.1 to 5% (4, 6, 9, 17–19). In our study, SRCC accounted for 1.20% of the colon cancer, which was consistent with the reported frequency. It was found that SRCC histology was more likely to be related to T4 stage, N2 stage, and grade III/IV, indicating that SRCC histology was more likely to be associated with adverse tumor pathology. SRCC was reported to be associated with peritoneal seeding and infiltration into lymphatics and nodes more frequently, which was attributed to the mucopolysaccharide nature of the colloid-type carcinoma which prevents discrimination of host immunocytes for tumor cells and thus allowing easier invasion into peri-intestinal tissue and subsequent lymphatics (4). Some previous studies indicated that primary colorectal signet-ring cell carcinoma tended to arise before 40 years of age (17, 20–22), and another series reported that the mean age varied from 52 to 67 years (6). The patients’ mean age of SRCC histology in the present study was 68.51 years, which was not entirely consistent with previous studies.

Although some researchers reported that SRCC had worse prognosis compared with AD (4, 5, 7–10), disputes about the prognostic value of histology of SRCC still existed (11–13, 23, 24) and clearly more research was required to solve this controversial issue. In the present study, it was found that SRCC histology was independently associated with 30.2% increased risk of colon cancer-specific mortality compared with AD histology in the whole cohort. In subgroup analyses, however, results of multivariate analyses showed that the CSS difference between AD and SRCC was not statistically significant in stage II colon cancer (P = 0.388) and SRCC histology had 14.5% increased risk of cancer-specific mortality with the borderline statistical significance compared with the histology of AD in stage III colon cancer. In 2014, it was reported that SRCC was an independent prognostic marker for a bad prognosis in stage III colon cancer (11). Recently, a retrospective analysis also found that SRCC did not negatively impact survival in stage II colon cancer after risk-adjusting for other prognostic factors (10). The different roles of SRCC histology in stage II and stage III colon cancer might partly explain the controversy about the prognosis of SRCC histology in colon cancer.

Further, the subgroup analyses showed that, in stage II colon cancer with the histology of AD, the CSS of colon cancer patients with the receipt of chemotherapy was similar to those without the receipt of chemotherapy though survival difference was statistically significant and the 5-year CSS rates of patients with and without the receipt of chemotherapy were 86.7 and 87.2%, respectively (P = 0.0078); after adjusting for many other factors that influenced the CSS of colon cancer, the receipt of chemotherapy had significantly 12.6% decreased risk of cancer-specific mortality (P < 0.001), showing that the receipt of chemotherapy independently improved CSS of stage II colon cancer with AD histology, which supported the chemotherapy use in stage II colon cancer reported by some previous studies (25, 26). In stage III colon cancer with the histology of AD, it was found that the CSS of colon cancer patients with the receipt of chemotherapy was significantly better than those without the receipt of chemotherapy and the 5-year CSS rates of patients with and without the receipt of chemotherapy were 77.5 and 64.4% (P < 0.001); after adjusting for many other factors that influenced the CSS of colon cancer, the receipt of chemotherapy had significantly 45.3% decreased risk of cancer-specific mortality, showing that chemotherapy use had greatly improved patient outcomes in stage III colon cancer, which was consistent with previous studies (27–29).

In stage II colon cancer with the histology of SRCC, however, it was found that the receipt of chemotherapy significantly decreased the CSS of colon cancer patients and the 5-year CSS rates of patients with and without the receipt of chemotherapy were 74.9 and 87.2%, respectively (P = 0.045); after adjusting for many other factors that influenced the CSS of colon cancer, the receipt of chemotherapy had significantly 70.00% increased risk of cancer-specific mortality. To the best of our knowledge, this was the first population-based study to focus on the efficacy of chemotherapy in stage II colon cancer with the histology of SRCC. Given the increased risk of colon cancer-specific mortality in stage II SRCC with the chemotherapy use, we held the view that chemotherapy should not be treated in stage II SRCC.

In stage III colon cancer with the histology of SRCC, it was found that the receipt of chemotherapy improved the CSS of colon cancer patients and the 5-year CSS rates of patients with and without the receipt of chemotherapy were 53.1 and 49.3%, respectively (P < 0.001), after adjusting for many other factors that influenced the CSS of colon cancer, the receipt of chemotherapy had significantly 24.6% decreased risk of cancer-specific mortality. Hugen et al. (11) reported that there was a comparable benefit from adjuvant chemotherapy in stage III AD and SRCC. In our study, combined the above analysis, however, we could find that SRCC had worse response to chemotherapy compared with AD in stage III colon cancer, we believed that it might account from the changes of treatment regimens during the past twenty years because cases selected in Hugen’s study were from as early as 1989. The receipt of chemotherapy could also significantly improve the prognosis of SRCC, therefore, we still recommended the chemotherapy use in stage III SRCC.

There were some potential weaknesses in our study. Firstly, some information including the chemotherapy regimens, chemotherapy duration, and basic diseases were not available for the included patients due to the limitation of SEER database. Secondly, our research was a retrospective type of study and the inherent deficiencies could lead to confusion or observer bias which cannot be removed. However, SRCC was a very rare disease, only large cancer database like SEER would be suitable for the investigation of it, we believed that the large size and breadth of this database across the US could mitigate the drawbacks outlined above.

## Conclusions

Using a large cancer database, we found that the CSS difference between AD and SRCC was not statistically significant in stage II colon cancer (P = 0.388) and SRCC histology had 14.5% increased risk of cancer-specific mortality with the borderline statistical significance compared with the histology of AD in stage III colon cancer. In addition, we provided the first compelling evidence that chemotherapy was associated with the increased risk of colon cancer-specific mortality and chemotherapy should not be treated in stage II SRCC. The receipt of chemotherapy could significantly improve the prognosis of stage III SRCC, therefore, we still recommended the chemotherapy use in stage III SRCC.

## Data Availability Statement

Publicly available data sets were analyzed in this study. The raw data supporting the conclusions of this article are available upon reasonable request from the corresponding author.

## Author Contributions

YW: concept and design. HJ and DS: collection and assembly of data. PZ: data analysis and interpretation. HJ: results interpretation and manuscript writing. HJ and DS: manuscript revision. All authors contributed to the article and approved the submitted version.

## Conflict of Interest

The authors declare that the research was conducted in the absence of any commercial or financial relationships that could be construed as a potential conflict of interest.

## Supplementary Material

Supplementary Figure 1 Flowchart for creation of the SEER patient dataset.
