# Supplementary figures and images for: The Predictive and Guidance Value of Signet Ring Cell Histology for Stage II/III Colon Cancer Response to Chemotherapy
Source: Front Oncol. 2021 Feb 23;11:631995. doi: 10.3389/fonc.2021.631995 (PMC7940524; doi:10.3389/fonc.2021.631995)

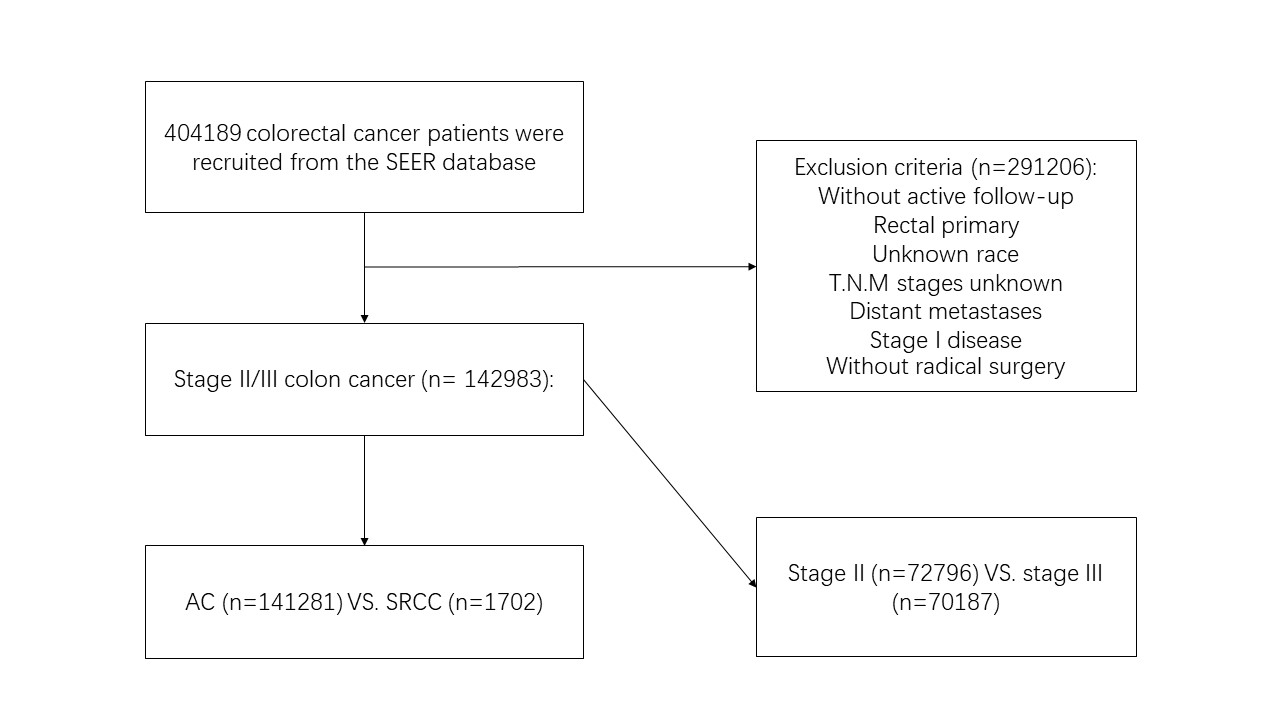

Supplement: Supplementary Figure 1 — Flowchart for creation of the SEER patient dataset. [file Image_1.jpeg]
