# Supplementary material for: The Predictive and Guidance Value of Signet Ring Cell Histology for Stage II/III Colon Cancer Response to Chemotherapy
Source: Front Oncol. 2021 Feb 23;11:631995. doi: 10.3389/fonc.2021.631995 (PMC7940524; doi:10.3389/fonc.2021.631995)
Supplement: Supplementary file 2 [file Table_1.docx]

**Table S1. Cox regression analyses of prognostic factors for CSS in stage II colon cancer.**

| **Variable** | **Univariate analyses** | |  | **Multivariate analyses** | |
| --- | --- | --- | --- | --- | --- |
|  | **HR (95%CI)** | ***P*** |  | **HR (95%CI)** | ***P*** |
| **T stage** |  | <0.001 |  |  | <0.001 |
| **T3** |  |  |  | 1 |  |
| **T4** |  |  |  | 2.786 (2.656-2.923) |  |
| **Age (years)** |  | <0.001 |  |  | <0.001 |
| **≤65** |  |  |  | 1 |  |
| **>65** |  |  |  | 1.697 (1.616-1.781) |  |
| **Race** |  | <0.001 |  |  | <0.001 |
| **White** |  |  |  | 1 |  |
| **Black** |  |  |  | 1.414 (1.331-1.502) | <0.001 |
| **Other** |  |  |  | 0.910 (0.937-0.990) | 0.028 |
| **Gender** |  | 0.262 |  |  |  |
| **Male** |  |  |  |  |  |
| **Female** |  |  |  |  |  |
| **Grade** |  | <0.001 |  |  | <0.001 |
| **Grade I/II** |  |  |  | 1 |  |
| **Grade III/IV** |  |  |  | 1.071 (1.015-1.129) | 0.012 |
| **Unknown** |  |  |  | 1.291 (1.129-1.477) | <0.001 |
| **Histology and chemotherapy** |  | 0.003 |  |  | <0.001 |
| **Adenocarcinoma, No/unknown** |  |  |  | 1.136 (0.851-1.516) | 0.388 |
| **Adenocarcinoma, Yes** |  |  |  | 0.993 (0.741-1.331) | 0.963 |
| **SRCC, No/unknown** |  |  |  | 1 |  |
| **SRCC, Yes** |  |  |  | 1.700 (1.032-2.801) | 0.037 |

**Table S2. Cox regression analyses of prognostic factors for CSS in stage III colon cancer.**

| **Variable** | **Univariate analyses** | |  | **Multivariate analyses** | |
| --- | --- | --- | --- | --- | --- |
|  | **HR (95%CI)** | ***P*** |  | **HR (95%CI)** | ***P*** |
| **T stage** |  | <0.001 |  |  | <0.001 |
| **T1** |  |  |  | 1 |  |
| **T2** |  |  |  | 1.444 (1.246-1.673) | <0.001 |
| **T3** |  |  |  | 3.019 (2.654-3.434) | <0.001 |
| **T4** |  |  |  | 6.182 (5.426-7.044) | <0.001 |
| **N stage** |  | <0.001 |  |  | <0.001 |
| **N1** |  |  |  | 1 |  |
| **N2** |  |  |  | 1.877 (1.821-1.935) |  |
| **Age (years)** |  | <0.001 |  |  | <0.001 |
| **≤65** |  |  |  | 1 |  |
| **>65** |  |  |  | 1.406 (1.362-1.452) |  |
| **Race** |  | <0.001 |  |  | <0.001 |
| **White** |  |  |  | 1 |  |
| **Black** |  |  |  | 1.292 (1.237-1.350) | <0.001 |
| **Other** |  |  |  | 0.947 (0.897-0.999) | 0.048 |
| **Gender** |  | 0.963 |  |  |  |
| **Male** |  |  |  |  |  |
| **Female** |  |  |  |  |  |
| **Grade** |  | <0.001 |  |  | <0.001 |
| **Grade I/II** |  |  |  | 1 |  |
| **Grade III/IV** |  |  |  | 1.259 (1.218-1.300) | <0.001 |
| **Unknown** |  |  |  | 1.189 (1.074-1.316) | 0.001 |
| **Histology and chemotherapy** |  | <0.001 |  |  | <0.001 |
| **Adenocarcinoma, No/unknown** |  |  |  | 0.874 (0.762-1.002) | 0.054 |
| **Adenocarcinoma, Yes** |  |  |  | 0.478 (0.417-0.548) | <0.001 |
| **SRCC, No/unknown** |  |  |  | 1 |  |
| **SRCC, Yes** |  |  |  | 0.754 (0.632-0.900) | 0.002 |
